# Aging effects on osteoclast progenitor dynamics affect variability in bone turnover via feedback regulation

**DOI:** 10.1093/jbmrpl/ziad003

**Published:** 2024-01-04

**Authors:** Young Kwan Kim, Yoshitaka Kameo, Sakae Tanaka, Taiji Adachi

**Affiliations:** Laboratory of Biomechanics, Department of Biosystems Science, Institute for Life and Medical Sciences, Kyoto University, Kyoto 606-8507, Japan; Department of Orthopaedic Surgery, Faculty of Medicine, The University of Tokyo, Tokyo 113-0033, Japan; Laboratory of Biomechanics, Department of Biosystems Science, Institute for Life and Medical Sciences, Kyoto University, Kyoto 606-8507, Japan; Department of Engineering Science and Mechanics, Shibaura Institute of Technology, Tokyo 135-8548, Japan; Department of Orthopaedic Surgery, Faculty of Medicine, The University of Tokyo, Tokyo 113-0033, Japan; Laboratory of Biomechanics, Department of Biosystems Science, Institute for Life and Medical Sciences, Kyoto University, Kyoto 606-8507, Japan

**Keywords:** osteoporosis, bone turnover, variability, progenitor, in silico experiment

## Abstract

Bone turnover markers (BTMs) are commonly used in osteoporosis treatment as indicators of cell activities of bone-resorbing osteoclasts and bone-forming osteoblasts. The wide variability in their values due to multiple factors, such as aging and diseases, makes it difficult for physicians to utilize them for clinical decision-making. The progenitors of osteoclasts and osteoblasts are indispensable for a comprehensive interpretation of the variability in BTM values because these upstream progenitors strongly regulate the downstream cell activities of bone turnover. However, understanding the complex interactions among the multiple populations of bone cells is challenging. In this study, we aimed to gain a fundamental understanding of the mechanism by which the progenitor dynamics affect the variability in bone turnover through in silico experiments by exploring the cell dynamics with aging effects on osteoporosis. Negative feedback control driven by the consumptive loss of progenitors prevents rapid bone loss due to excessive bone turnover, and through feedback regulation, aging effects on osteoclast differentiation and osteoclast progenitor proliferation cause variability in the osteoclast and osteoblast activity balance and its temporal transition. By expressing the variability in the bone turnover status, our model describes the individualities of patients based on their clinical backgrounds. Therefore, our model could play a powerful role in assisting tailored treatment and has the potential to resolve the various health problems associated with osteoporosis worldwide.

## Teaser

Considering interaction of upstream progenitors and downstream bone-resorbing and bone-forming cells in bone cell lineages is indispensable for a comprehensive interpretation of bone turnover. However, understanding the complex interactions among the multiple populations of bone cells is challenging. Here, we aimed to gain a fundamental understanding of the mechanism by which the progenitor cell dynamics affect variability in bone turnover through in silico experiments. A model incorporating the upstream and downstream dynamics of bone cell lineages showed that consumptive loss of progenitors works as negative feedback to prevent excessive bone turnover. It was demonstrated that, via this feedback regulation, aging effects on the cell genesis of bone-resorbing cells and the proliferation of their progenitors affect the variability in turnover balance of bone resorption and formation and its temporal transition.

## Graphical Abstract

**Graphical Abstract:**
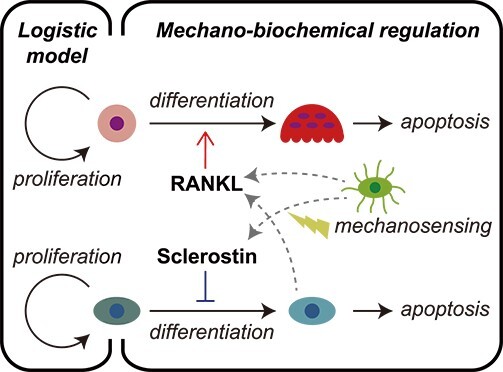

## Introduction

Osteoporosis is a common metabolic bone disease caused by an abnormal bone turnover that results in bone loss over time.^1^ Excessive bone loss leads to fragility fractures, and subsequent loss of mobility puts an enormous burden on the economy and patient health worldwide.^2-5^ In the clinical practice of osteoporosis, proper assessment of the bone turnover status is essential to realize an appropriate treatment for individual patients.

Bone turnover markers (BTMs), such as C-terminal telopeptides of type I collagen and N-terminal propeptides of type I procollagen, are commonly used in osteoporosis clinical practice as indicators of a patient’s bone turnover status based on the activities of bone-resorbing osteoclasts and bone-forming osteoblasts.^6^ Although BTMs are expected to be useful bone biomarkers for predicting fracture risk and monitoring therapeutic efficacy in osteoporosis treatment,^7^ a wide variability in their values due to multiple factors, such as aging and diseases, makes it difficult for physicians to utilize them for clinical decision-making.^8-10^

The progenitors of osteoclasts and osteoblasts are indispensable for comprehensively interpreting this variability in BTM values because these upstream progenitors strongly regulate the downstream cell activities of bone turnover and the progenitor dynamics are also affected by multiple factors.^11-14^ However, understanding the complex interactions in multiple populations of bone cells is extremely challenging.

In this study, we aimed to gain a fundamental understanding of the mechanism by which osteoclast and osteoblast progenitor dynamics affect the variability in bone turnover through in silico experiments. Although there are a variety of clinical and systemic factors that contribute to bone turnover, such as vitamin D levels, calcium levels, gut microbiome,^15^^,^^16^ and use of pharmaceuticals, here, we focused on progenitor dynamics and explored how bone turnover in age-related osteoporosis is regulated by interactions among multiple populations of bone cells beyond their lineage. In order to understand the fundamental mechanism of variability in bone turnover in terms of progenitor regulation, we perturbed the number of osteoclast progenitors and the concentration of receptor activator of nuclear factor-κB ligand (RANKL, i.e., a main regulator of osteoclastogenesis) as representative effects of aging on both upstream and downstream bone cell lineages. Given that the osteoclast and osteoblast activities are believed to be regulated by osteocyte mechanosensing, we assumed an osteoporosis model in which bone adaptation to mechanical status is impaired due to age-related effects on bone turnover, resulting in bone loss. It has been demonstrated that the consumptive loss of progenitors could work as a negative feedback to prevent excessive bone turnover and that, via feedback regulation, aging effects on osteoclast differentiation and osteoclast progenitor proliferation cause variability in the bone turnover balance and its temporal transition.

## Materials and methods

### In silico experimental platform

**Figure 1:**
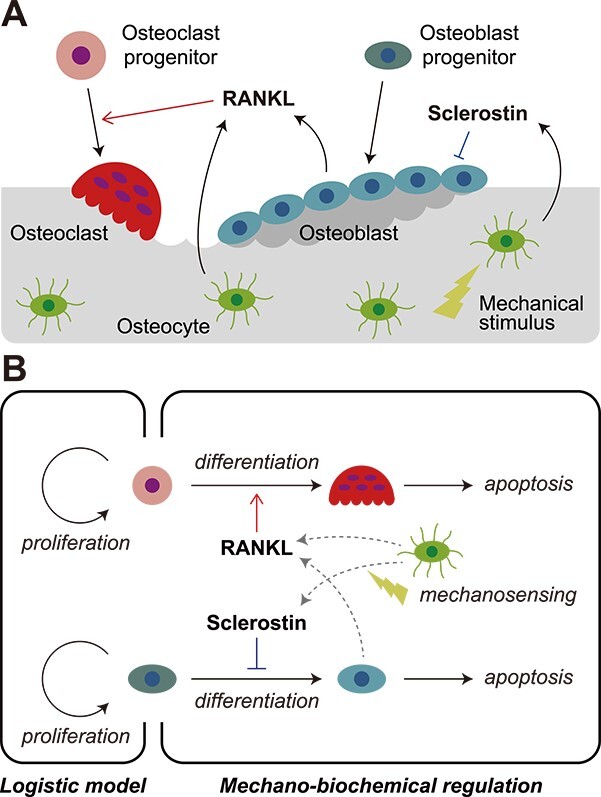
Modeling of bone cell dynamics that incorporate multi-population interactions. (A) Overview of bone cell activities regulated by mechanical and biochemical factors. RANKL, receptor activator of nuclear factor-κB ligand. (B) Incorporating dynamics of the upstream progenitors expressed by a logistic model and the downstream osteoclasts and osteoblasts differentiated by mechano-biochemical regulation.

A mathematical model is proposed in this study based on a previously developed in silico experimental platform (V-Bone) that enables the 4-dimensional observation of bone morphological changes and bone cell activities.^17^ The V-Bone models mechano-biochemical regulation, in which osteocytes embedded in the bone matrix sense mechanical stimuli and orchestrate bone-resorbing osteoclasts and bone-forming osteoblasts on the bone surface via intercellular signaling (Figure 1A). The sclerostin (SCL), secreted by osteocytes, inhibits osteoblastogenesis, and its expression is reduced by mechanical loading.^18^^,^^19^ The RANKL, secreted by osteoblasts and osteocytes, is a key signaling molecule that induces osteoclastogenesis by binding to its receptor, RANK.^20^^,^^21^ In our model, the stress distribution in the bone matrix analyzed using the voxel-based finite element (FE) method determines the distribution of SCL, which affects the distribution of signaling molecules, such as RANKL and osteoprotegerin. The spatial and temporal behaviors of the signaling molecules were analyzed by solving the reaction-diffusion equations of the molecules. Furthermore, the probability of differentiation or apoptosis of osteoclasts and osteoblasts was determined based on the values of stress and the concentration of signaling molecules on the bone surface. Osteoclast and osteoblast activities were assumed to be driven to change bone morphology toward the acquisition of mechanical stability. For details of the in silico model, please refer to the previous work by Kameo et al.^17^

### Modeling of bone cell dynamics that incorporate multi-population interactions

To investigate the mechanism by which the progenitors of osteoclasts and osteoblasts affect the variability in bone turnover, we modeled bone cell dynamics by incorporating multi-population interactions of upstream progenitor cells and downstream osteoclasts and osteoblasts (Figure 1B). Progenitor dynamics were modeled by considering the proliferation of progenitors and their consumption by differentiation into downstream cells. More specifically, the time evolution equations of the progenitor number density were assumed to consist of a proliferation term expressed by a logistic model^22^ and a consumption term determined according to the number density of downstream cells differentiated by mechano-biochemical regulation, as follows:


(1) \begin{equation*} \frac{d{N}_{\mathrm{OC}\ \mathrm{prog}}}{dt}={r}_{\mathrm{OC}\ \mathrm{prog}}^{\mathrm{prolif}}{N}_{\mathrm{OC}\ \mathrm{prog}}\left(1-\frac{N_{\mathrm{OC}\ \mathrm{prog}}}{N_{\mathrm{OC}\ \mathrm{prog}}^{\mathrm{max}}}\right)-{r}_{\mathrm{OC}}^{\mathrm{diff}}{N}_{\mathrm{OC}} \end{equation*}

and


(2) \begin{equation*} \frac{d{N}_{\mathrm{OB}\ \mathrm{prog}}}{dt}={r}_{\mathrm{OB}\ \mathrm{prog}}^{\mathrm{prolif}}{N}_{\mathrm{OB}\ \mathrm{prog}}\left(1-\frac{N_{\mathrm{OB}\ \mathrm{prog}}}{N_{\mathrm{OB}\ \mathrm{prog}}^{\mathrm{max}}}\right)-{r}_{\mathrm{OB}}^{\mathrm{diff}}{N}_{\mathrm{OB}}, \end{equation*}

where ${N}_{\mathrm{OC}}$, ${N}_{\mathrm{OB}}$, ${N}_{\mathrm{OC}\ \mathrm{prog}}$, and ${N}_{\mathrm{OB}\ \mathrm{prog}}$ denote the number densities of osteoclasts, osteoblasts, osteoclast progenitors, and osteoblast progenitors, respectively. The ${N}_{\mathrm{OC}\ \mathrm{prog}}^{\mathrm{max}}$ and ${N}_{\mathrm{OB}\ \mathrm{prog}}^{\mathrm{max}}$denote the maximum number density of progenitors, representing the carrying capacity in the bone marrow environment. The rates ${r}_{\mathrm{OC}\ \mathrm{prog}}^{\mathrm{prolif}}$ and ${r}_{\mathrm{OB}\ \mathrm{prog}}^{\mathrm{prolif}}$ denote the cell proliferation rates of each progenitor, and ${r}_{\mathrm{OC}}^{\mathrm{diff}}$ and ${r}_{\mathrm{OB}}^{\mathrm{diff}}$ denote the cell differentiation rates into osteoclasts and osteoblasts, respectively.

The V-Bone models the probability of cell genesis of osteoclasts and osteoblasts (${p}_{\mathrm{gen}}^{\mathrm{OC}}$ and ${p}_{\mathrm{gen}}^{\mathrm{OB}}$) based on biochemical factors by using activator and repressor functions of the concentration of signaling molecules.^17^ In the proposed model, in addition to biochemical regulation, ${p}_{\mathrm{gen}}^{\mathrm{OC}}$ and ${p}_{\mathrm{gen}}^{\mathrm{OB}}$ were assumed to be reduced depending on the number density of each progenitor cell, as follows:


(3) \begin{equation*} {p}_{\mathrm{gen}}^{\mathrm{OC}}=\frac{N_{\mathrm{OC}\ \mathrm{prog}}^n}{K_{\mathrm{OC}\ \mathrm{prog}}^n+{N}_{\mathrm{OC}\ \mathrm{prog}}^n}{\left.{p}_{\mathrm{gen}}^{\mathrm{OC}}\right|}_{\mathrm{Biochem}} \end{equation*}

and


(4) \begin{equation*} {p}_{\mathrm{gen}}^{\mathrm{OB}}=\frac{N_{\mathrm{OB}\ \mathrm{prog}}^n}{K_{\mathrm{OB}\ \mathrm{prog}}^n+{N}_{\mathrm{OB}\ \mathrm{prog}}^n}{\left.{p}_{\mathrm{gen}}^{\mathrm{OB}}\right|}_{\mathrm{Biochem}}, \end{equation*}

where ${\left.{p}_{\mathrm{gen}}^{\mathrm{OC}}\right|}_{\mathrm{Biochem}}$ and ${\left.{p}_{\mathrm{gen}}^{\mathrm{OB}}\right|}_{\mathrm{Biochem}}$ are the probabilities of cell genesis considering only biochemical factors, ${K}_{\mathrm{OC}\ \mathrm{prog}}$ and ${K}_{\mathrm{OB}\ \mathrm{prog}}$ are the activation constants, and *n* is the Hill coefficient that determines the steepness of the sigmoidal curve.^23^

### A 3D image-based FE model of cancellous bone

Mechanical stimuli in the bone matrix were analyzed using the voxel FE method. An FE model of the cancellous bone was constructed from X-ray micro-CT images of a swine femoral head based on a previous procedure reported by Kim et al.^24^ The model was constructed as a cube of 45 × 45 × 45 voxels, and each element was a cubic voxel with an edge size of 24 μm. The bone was assumed to be a homogeneous and isotropic linear elastic material with a Young’s modulus *E* = 20 GPa and a Poisson’s ratio ν = 0.3. Uniform compressive stresses were applied to the cancellous FE model. For details of the boundary conditions, please refer to the previous procedure.^24^

### In silico experiment of age-related osteoporosis

To examine how progenitor dynamics affect the variability in bone turnover during aging, we performed in silico experiments for 100 weeks by perturbing the effects of aging on both upstream and downstream bone cell lineages. The RANKL, which induces osteoclastogenesis, increases with aging.^25^^,^^26^ Based on the previous reports that RANKL expression is enhanced by SCL,^27^^,^^28^ the rate of RANKL production ${P}_{\mathrm{RANKL}}$ was described by using the activator function of ${\phi}_{\mathrm{SCL}}$ as follows:


(5) \begin{equation*} {P}_{\mathrm{RANKL}}={\beta}_{\mathrm{RANKL}}\frac{\phi_{\mathrm{SCL}}^n}{K_{\mathrm{SCL}}^n+{\phi}_{\mathrm{SCL}}^n}, \end{equation*}

where ${\beta}_{\mathrm{RANKL}}$ is the maximum production rate of RANKL, ${K}_{\mathrm{SCL}}$ is the activation constant, and ${\phi}_{\mathrm{SCL}}$ is the SCL concentration. In our experiments, ${\beta}_{\mathrm{RANKL}}$ was perturbed to express the aging effects on RANKL.

Previous studies have shown that the number of osteoclast progenitor cells increases with aging.^12^^,^^29-31^ In our experiments, ${r}_{\mathrm{OC}\ \mathrm{prog}}^{\mathrm{prolif}}$ was perturbed to express the aging effects on the osteoclast progenitor number in eqn (1). The parameters used in the in silico experiments are listed in Supplementary Tables S1–S3.

## Results

### Activated bone turnover due to age-related RANKL increase is attenuated by negative feedback via consumption of osteoclast progenitors

**Figure 2:**
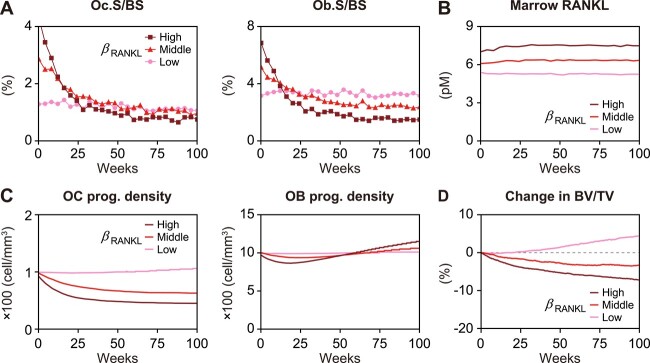
Activated bone turnover due to age-related RANKL increase is attenuated by negative feedback via consumption of osteoclast progenitors. (A) Osteoclast and osteoblast activities with aging effects on RANKL. Aging effects were set as low, middle, and high ${\beta}_{\mathrm{RANKL}}$ (=0.24, 0.28, and 0.32 pM/s). (B) Concentration of RANKL in the bone marrow with aging effects. (C) Density of the progenitor cell number in the bone marrow. (D) % change in bone volume from the baseline. The gray dotted line is the baseline representing bone volume at 0 weeks. RANKL, receptor activator of nuclear factor-κB ligand; Oc.S/BS, osteoclast surface/bone surface; Ob.S/BS, osteoblast surface/bone surface; OC prog., osteoclast progenitor; OB prog., osteoblast progenitor; BV/TV, bone volume/tissue volume.

We first investigated the effects of age-related RANKL increase on bone turnover. After preliminary parameter sensitivity analyses, we set the parameter values at which progenitor dynamics were almost constant, assuming a young state with no effect of aging. Under these parameter settings, we conducted in silico experiments by perturbing the maximum RANKL production rate ${\beta}_{\mathrm{RANKL}}$ for 100 weeks with the osteoclast progenitor proliferation rate ${r}_{\mathrm{OC}\ \mathrm{prog}}^{\mathrm{prolif}}$ fixed. During the first 20 weeks, osteoclast differentiation was strongly promoted (Figure 2A, left panel) by an increase in RANKL due to the aging effect (Figure 2B), and osteoblast differentiation was promoted in a coupled manner with osteoclast differentiation (Figure 2A, right panel). In parallel, the number of osteoclast progenitors was significantly decreased in contrast to moderate changes in the number of osteoblast progenitors (Figure 2C). This significant decrease in osteoclast progenitors was due to excessive consumption by strongly promoting osteoclast differentiation, as the osteoclast progenitor proliferation rate ${r}_{\mathrm{OC}\ \mathrm{prog}}^{\mathrm{prolif}}$ was fixed. The reduction in osteoclast progenitors weakened the promotion of osteoclast and subsequent osteoblast differentiation, causing low bone turnover after 25 weeks (Figure 2A). As a result, bone volume loss was rapid due to high turnover in the early period, whereas it became slower closer to 25 weeks due to low turnover (Figure 2D). It has been shown that activated bone turnover in aging is attenuated by the negative feedback control triggered by the consumption of osteoclast progenitors, which are upstream cells in the osteoclast lineage.

### Age-related enhancement of osteoclast progenitor proliferation maintains high bone turnover with osteoclast dominance

**Figure 3:**
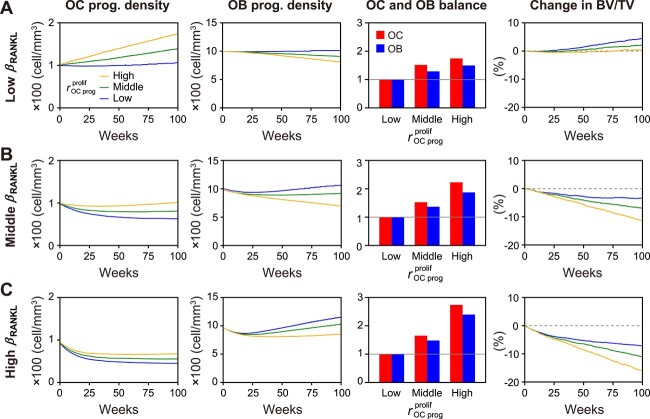
Age-related enhancement of osteoclast progenitor proliferation maintains high bone turnover with osteoclast dominance. (A–C) Comparison of density of the progenitor cell number (first and second panels from left), the balance between osteoclast and osteoblast activities (third panels), and % change in bone volume from the baseline (fourth panels) with aging effects on osteoclast progenitor proliferation among RANKL conditions. In each condition of ${\beta}_{\mathrm{RANKL}}$ (=0.24, 0.28, and 0.32 pM/s), aging effects were set as a low, middle, and high rate of osteoclast progenitor proliferation ${r}_{\mathrm{OC}\ \mathrm{prog}}^{\mathrm{prolif}}$ (=0.002, 0.003, and 0.004/mm^3^/day). In the graphs of OC and OB balance (third panel), each bar represents OC or OB activity, shown as its normalized value that was calculated as the ratio of Oc.S or Ob.S with each ${r}_{\mathrm{OC}\ \mathrm{prog}}^{\mathrm{prolif}}$ (=0.002, 0.003, and 0.004/mm^3^/day) to that of the low ${r}_{\mathrm{OC}\ \mathrm{prog}}^{\mathrm{prolif}}$ (=0.002/mm^3^/day). RANKL, receptor activator of nuclear factor-κB ligand; OC, osteoclast; OB, osteoblast; OC prog., osteoclast progenitor; OB prog., osteoblast progenitor. Oc.S, osteoclast surface; Ob.S, osteoblast surface; BV/TV, bone volume/tissue volume.

We further investigated the effects of the age-related proliferation of osteoclast progenitors on bone turnover accompanied by negative feedback via the consumption of osteoclast progenitors. By expanding the simple perturbation of RANKL in the previous section, we conducted in silico experiments with perturbation of the osteoclast progenitor proliferation rate ${r}_{\mathrm{OC}\ \mathrm{prog}}^{\mathrm{prolif}}$ for each aging condition of the maximum RANKL production rate ${\beta}_{\mathrm{RANKL}}$. Under all ${\beta}_{\mathrm{RANKL}}$ conditions, as ${r}_{\mathrm{OC}\ \mathrm{prog}}^{\mathrm{prolif}}$ was more enhanced by the aging effect, osteoclast differentiation and subsequent osteoblast differentiation were promoted (Supplementary Figure S1). Under the low ${\beta}_{\mathrm{RANKL}}$ condition, osteoclast progenitors showed stable or increasing trends, and osteoblast progenitors showed stable or moderately decreasing trends over time (Figure 3A, first and second panels from left). Under higher ${\beta}_{\mathrm{RANKL}}$conditions, progenitors of osteoclasts and osteoblasts showed decreasing trends in the early period (Figure 3B and C, first and second panels) due to consumption by promoted osteoclast and osteoblast differentiation (Supplementary Figure S1B and C). However, with the osteoclast progenitor dynamics (Figure 3B and C, first panels), as ${r}_{\mathrm{OC}\ \mathrm{prog}}^{\mathrm{prolif}}$ increased, these decreasing trends in the early period were alleviated. By contrast, the decreasing trend in osteoblast progenitors was not alleviated because there was no enhancement in their proliferation owing to aging (Figure 3B and C, second panels).

To examine how these contrasting dynamics of each progenitor induced by aging affect the balance between osteoclast and osteoblast activities, we compared their activities at 100 weeks. Under high ${r}_{\mathrm{OC}\ \mathrm{prog}}^{\mathrm{prolif}}$ conditions, osteoclast activity was greater than osteoblast activity under all ${\beta}_{\mathrm{RANKL}}$conditions (Figure 3A–C, third panels). Furthermore, as the ${r}_{\mathrm{OC}\ \mathrm{prog}}^{\mathrm{prolif}}$ was more enhanced, the difference in the activities of osteoclasts and osteoblasts increased. As a result, as both ${\beta}_{\mathrm{RANKL}}$and ${r}_{\mathrm{OC}\ \mathrm{prog}}^{\mathrm{prolif}}$ increased, the bone volume was greatly reduced (Figure 3A–C, fourth panels). It has been suggested that the negative feedback via the consumption of the progenitors is suppressed for osteoclasts by the age-related enhancement of ${r}_{\mathrm{OC}\ \mathrm{prog}}^{\mathrm{prolif}}$, but not for osteoblasts, and this discrepancy could maintain high turnover in a balance of osteoclast dominance, thereby resulting in a significant bone loss.

### Aging effects cause variability in the osteoclast and osteoblast activity balance and its temporal transition

**Figure 4:**
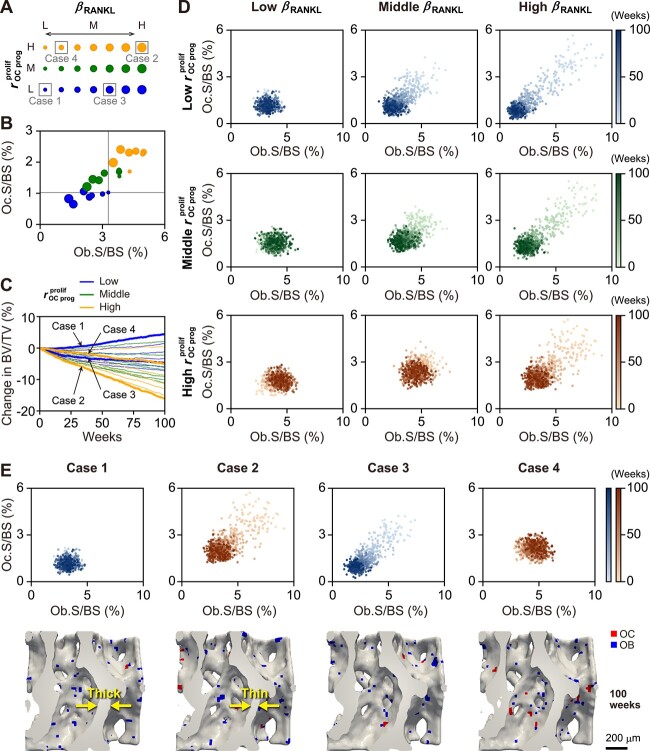
Aging effects cause variability in the osteoclast and osteoblast activity balance and its temporal transition. (A) Aging effects on RANKL and the osteoclast progenitor proliferation rate. Each row of circles represents an aging condition of ${r}_{\mathrm{OC}\ \mathrm{prog}}^{\mathrm{prolif}}$, which was set as 0.002 (L, low), 0.003 (M, middle), and 0.004 (H, high)/mm^3^/day. Each circle size represents an aging condition of ${\beta}_{\mathrm{RANKL}}$; ${\beta}_{\mathrm{RANKL}}$ was set as seven perturbed values ranging from 0.24 to 0.32 pM/s in an equally spaced manner. (B) Cell activity balance in the final period. The averaged cell activities in the last 4 weeks of the 100-week observation was plotted. Each plot corresponds to each aging condition in (A). The gray lines represent the reference status with the lowest aging effects on both ${\beta}_{\mathrm{RANKL}}$ and ${r}_{\mathrm{OC}\ \mathrm{prog}}^{\mathrm{prolif}}$. (C) % change in bone volume from the baseline. Cases 1–4 are characteristic cases chosen based on the bone volume at 100 weeks and are emphasized with thick lines. (D) Comparison of the osteoclast and osteoblast activity balance and its temporal transition among aging conditions. Representative conditions were shown here with ${\beta}_{\mathrm{RANKL}}$ = 0.24 (low), 0.28 (middle), and 0.32 (high) pM/s and with ${r}_{\mathrm{OC}\ \mathrm{prog}}^{\mathrm{prolif}}$ = 0.002 (low), 0.003 (middle), and 0.004 (high)/mm^3^/day. Each plot represents daily observed cell activity. Each color bar represents the elapsed time at which cell activity was observed, ranging from 0 to 100 weeks. (E) Comparison of the osteoclast and osteoblast activity balance and its temporal transition (upper panel) and bone morphology with surface cell activity at 100 weeks (lower panel) among the 4 cases. In the lower panels, osteoclasts and osteoblasts are shown as red and blue surface voxels (voxel size = 24 μm, scale bar = 200 μm). RANKL, receptor activator of nuclear factor-κB ligand; OC, osteoclast; OB, osteoblast; Oc.S/BS, osteoclast surface/bone surface; Ob.S/BS, osteoblast surface/bone surface; BV/TV, bone volume/tissue volume.

To understand the effect of aging on bone turnover variability, we investigated the balance of osteoclast and osteoblast activity with diverse aging effects on the maximum RANKL production rate ${\beta}_{\mathrm{RANKL}}$and osteoclast progenitor proliferation rate ${r}_{\mathrm{OC}\ \mathrm{prog}}^{\mathrm{prolif}}$ (Figure 4A). Comparison of the average cell activity balance in the final period (Figure 4B) showed that as ${r}_{\mathrm{OC}\ \mathrm{prog}}^{\mathrm{prolif}}$ increased, both osteoclast and osteoblast activities were promoted, as shown in Figure 3A–C. For each condition of ${r}_{\mathrm{OC}\ \mathrm{prog}}^{\mathrm{prolif}}$, as ${\beta}_{\mathrm{RANKL}}$ increased, the bone turnover tended to shift to a lower turnover, which represents stronger negative feedback via more excessive consumption induced by the ${\beta}_{\mathrm{RANKL}}$ increase. However, osteoclasts are less likely to be attenuated because the proliferation of osteoclast progenitors is enhanced by aging. Thus, the variability in the osteoclast and osteoblast balance was dependent on the diversity of aging effects, and such variability resulted in various patterns of changes in bone volume over time (Figure 4C).

We further investigated the temporal transition of the cell activity balance with the diverse aging effects (Figure 4D). For each condition of ${r}_{\mathrm{OC}\ \mathrm{prog}}^{\mathrm{prolif}}$ (see row), as ${\beta}_{\mathrm{RANKL}}$ increased, the bone turnover was initially more activated, but it was attenuated over time. For each condition of ${\beta}_{\mathrm{RANKL}}$ (see column), as ${r}_{\mathrm{OC}\ \mathrm{prog}}^{\mathrm{prolif}}$ increased, the attenuation of bone turnover was more alleviated and a higher turnover status was maintained. Diverse effects of aging on RANKL production and osteoclast progenitor proliferation result in variability in the balance of osteoclast and osteoblast activities and its temporal transition.

To understand the clinical importance of this variability due to diverse aging effects, we chose 4 characteristic cases from all conditions shown in Figure 4A based on the bone volume at 100 weeks (Figure 4C). For each case, the pattern of temporal transition in bone turnover and resultant bone morphology with surface cell activity are shown in Figure 4E. Cases 1 and 2 were the 2 contrasting cases with the highest and lowest bone volumes at 100 weeks, respectively. Case 1, with the lowest aging effects on both ${\beta}_{\mathrm{RANKL}}$ and ${r}_{\mathrm{OC}\ \mathrm{prog}}^{\mathrm{prolif}}$, showed a persistently low bone turnover from the beginning to the end of the observation period, resulting in the greatest bone gain with thick trabeculae. By contrast, case 2, with the highest aging effect on both ${\beta}_{\mathrm{RANKL}}$ and ${r}_{\mathrm{OC}\ \mathrm{prog}}^{\mathrm{prolif}}$, resulted in the greatest bone loss with thin trabeculae. In case 2, the turnover was initially intense, but it was attenuated by negative feedback over time, resulting in an osteoclast-dominant high-turnover status. Cases 3 and 4 were apparently similar, showing the same bone volume at 2 time points: 0 and 100 weeks. In case 3, where the aging effects were moderate on ${\beta}_{\mathrm{RANKL}}$ and lowest on ${r}_{\mathrm{OC}\ \mathrm{prog}}^{\mathrm{prolif}}$, the turnover was initially high, but it was strongly attenuated over time. In case 4, where the aging effects were low on ${\beta}_{\mathrm{RANKL}}$ but highest on ${r}_{\mathrm{OC}\ \mathrm{prog}}^{\mathrm{prolif}}$, a high bone turnover was maintained without negative feedback. Importantly, it seemed that the future bone volume after 100 weeks reduced faster in case 4 than in case 3 because of the distinct difference in bone turnover in the last observation period, which could also be predicted by the different rates of bone volume change in the latter half of the observation period (Figure 4C).

## Discussion

In this study, we aimed to gain a fundamental understanding of the mechanism that causes variability in bone turnover based on the interaction of upstream cells (the progenitors of osteoclasts and osteoblasts) and downstream cells (osteoclasts and osteoblasts) in bone cell lineages. In the in silico experiments, under low RANKL conditions, osteoclasts decreased and osteoclast precursors increased in number compared to those under high RANKL conditions. Similar results were reported in a previous in vivo study in mice, in which it was found that RANKL inhibition by an anti-RANKL antibody led to an increase in the immediate precursors of osteoclasts in the macrophage/monocyte lineage.^32^ Together with these experimental findings, our in silico results suggest that RANKL inhibition induces an increase in osteoclast precursors via osteoclast suppression. Moreover, from the viewpoint of aging effects on osteoporosis, it was found that negative feedback via the consumption of upstream progenitors was suppressed for osteoclasts, but not for osteoblasts, thereby maintaining a high bone turnover in osteoclast-dominant balance and resulting in a significant bone loss. It has been demonstrated that, via feedback regulation, aging effects on progenitors and RANKL cause variability in the balance of the osteoclast and osteoblast activities and its temporal transition.

These results suggest that the development of novel biomarkers for progenitor cell activity could provide a breakthrough in osteoporosis treatment. The BTMs, which are biomarkers of bone turnover used in clinical practice, are not always easy to interpret because they only provide instantaneous values at the time of blood sampling and can fluctuate depending on various factors. The novel biomarkers of the progenitors, when combined with BTMs, could enable not only the assessment of the current bone turnover status but also simultaneously predict its long-term change, thereby realizing a proactive therapy to make prompt decisions on initiating treatment and switching therapies. Therefore, the proposed biomarkers could be of tremendous clinical and social values by solving a significant medical and economic problem in aging societies worldwide through the prevention and early remission of osteoporosis.

Our model, which considers progenitor dynamics, can be applied to understand the bone turnover status of patients from various clinical backgrounds. For example, glucocorticoid administration, a cause of secondary osteoporosis, induces osteoclast activity by directly increasing osteoclast progenitors.^14^ Moreover, estrogen, whose loss causes postmenopausal osteoporosis, has been reported to suppress osteoclast activity by inhibiting the differentiation of osteoclast progenitors into osteoclasts^11^ and inducing the apoptosis of osteoclast progenitors.^13^ Our model has the potential to describe the various individualities of patients based on their clinical backgrounds and thereby could play a powerful role in providing a tailored treatment according to the individualities.

Our model contains simplified assumptions regarding unclarified mechanisms. For example, we expressed the relationship between RANKL production rate and SCL concentration in the bone marrow using the Hill function to describe the dependency between these signaling molecules. However, this relationship is not necessarily sigmoidal or even monotonically increasing or decreasing. If the relationship between SCL and RANKL is further clarified experimentally, it will be necessary to develop a model that accurately represents these findings. Moreover, to capture the essence of the complex dynamics of a broad spectrum of cell lineages, we modeled the cell dynamics of the upstream and downstream cells. The results of this simplified model indicate, in theory, that the feedback mechanism from upstream cells can affect the variability in bone metabolism. There are published studies in which the dynamics of upstream cells (e.g., accumulation and depletion) have been associated with clinically important issues in the treatment of osteoporosis, such as rapid bone loss after discontinuation of denosumab (an anti-RANKL antibody),^32^^,^^33^ and attenuation of bone formation markers after starting romosozumab (an anti-SCL antibody).^34^^,^^35^ To determine whether the feedback mechanism is associated with such clinical phenomena, cell lineage dynamics should be modeled in more detail. Such continuous efforts to refine the model through experimental studies will contribute to a better understanding of bone metabolism and resolve clinical issues in the treatment of osteoporosis.

In addition to understanding of cell number dynamics in aging, which we gained in this study, considering age-related impairment of cell function such as the cellular senescence due to specifically altered gene expression pattern^36-39^ is crucial for a thorough understanding of the mechanisms of variability in bone turnover in aging. The simultaneous examination of the effects of aging on cell function and cell number dynamics is challenging because of their complexity. This complexity underscores the value of in silico experiments and the need to continue this research. To accomplish this, it is important to consider the senescence of specific functions of bone cells, such as mechanosensing by osteocytes, and bone resorption and formation by osteoclasts and osteoblasts. A comprehensive model that takes into account these processes would provide valuable insights concerning the progression of skeletal aging.

A feedback mechanism is a fundamental function by which a biological system self-regulates the homeostasis of its physiological states.^40-44^ Bone homeostasis is thought to stabilize the bone shape through a mechano-biochemical feedback mechanism by which osteocytes produce and transmit signaling molecules based on mechanical stimuli to control resorption by osteoclasts and formation by osteoblasts.^45-47^ In silico studies of bone metabolism have described a pathological process in which the breakdown of this feedback system due to an abnormal bone turnover balance leads to significant bone loss.^17^^,^^24^^,^^48-51^ In this study, we further demonstrated that the feedback system associated with the consumptive loss of progenitors is another homeostatic mechanism that prevents rapid bone loss owing to abnormal bone turnover. Thus, our model enables a comprehensive understanding of bone metabolism, simultaneously based on the interaction across the lineage (i.e., between osteoclasts and osteoblasts) and the interaction within the lineage (i.e., between upstream progenitors and downstream differentiated cells). We believe that our biofidelic model will help clarify the pathogenesis of various types of osteoporosis and explore their treatment, thereby solving the adverse effects of osteoporosis that plague people around the world.

## Author contributions

Young Kwan Kim (Conceptualization, Data curation, Formal analysis, Funding acquisition, Investigation, Methodology, Project administration, Software, Validation, Visualization, Writing—original draft), Yoshitaka Kameo (Methodology, Software, Writing—review & editing), Sakae Tanaka (Conceptualization, Writing—review & editing), and Taiji Adachi (Conceptualization, Funding acquisition, Methodology, Project administration, Resources, Supervision, Writing—review & editing)

All authors approved the final version of the submitted manuscript.

## Funding

This work was supported by Japan Society for the Promotion of Science (JSPS KAKENHI: JP20K18020, JP23K15739, and JP20H00659), Japan Science and Technology Agency (JST-CREST: JPMJCR22L5), and the Cooperative Research Program (Joint Usage/Research Center Program) of the Institute for Life and Medical Sciences, Kyoto University.

## Conflicts of interest

None declared.

## Data availability

The data that support the findings of this study are available from the corresponding author upon reasonable request.

## Supplementary Material

ProgenitorJ_Supplemental_Materials_ziad003

## References

[1] Compston JE, McClung MR, Leslie WD. Osteoporosis. Lancet. 2019;393(10169):364–376. 10.1016/S0140-6736(18)32112-3. 30696576

[2] Orimo H, Yaegashi Y, Hosoi T, et al. Hip fracture incidence in Japan: estimates of new patients in 2012 and 25-year trends. Osteoporos Int. 2016;27(5):1777–1784. 10.1007/s00198-015-3464-8. 26733376 PMC4873530

[3] Borgstrom F, Karlsson L, Ortsater G, et al. International osteoporosis foundation, fragility fractures in Europe: burden, management and opportunities. Arch Osteoporos. 2020;15(1):59. 10.1007/s11657-020-0706-y. 32306163 PMC7166207

[4] Lewiecki EM, Chastek B, Sundquist K, et al. Osteoporotic fracture trends in a population of US managed care enrollees from 2007 to 2017. Osteoporos Int. 2020;31(7):1299–1304. 10.1007/s00198-020-05334-y. 32062687 PMC7280339

[5] Chandran M, Brind'Amour K, Fujiwara S, et al. Prevalence of osteoporosis and incidence of related fractures in developed economies in the Asia Pacific region: a systematic review. Osteoporos Int. 2023;34(6):1037–1053. 10.1007/s00198-022-06657-8. 36735053 PMC10202996

[6] Naylor K, Eastell R. Bone turnover markers: use in osteoporosis. Nat Rev Rheumatol. 2012;8(7):379–389. 10.1038/nrrheum.2012.86. 22664836

[7] Vasikaran S, Eastell R, Bruyere O, et al. IOF-IFCC Bone Marker Standards Working Group, markers of bone turnover for the prediction of fracture risk and monitoring of osteoporosis treatment: a need for international reference standards. Osteoporos Int. 2011;22(2):391–420. 10.1007/s00198-010-1501-1. 21184054

[8] Seibel MJ . Biochemical markers of bone turnover: part I: biochemistry and variability. Clin Biochem Rev. 2005;26(4):97–122. 16648882 PMC1320175

[9] Szulc P, Naylor K, Hoyle NR, Eastell R, Leary ET. National Bone Health Alliance Bone Turnover Marker Project, use of CTX-I and PINP as bone turnover markers: National Bone Health Alliance recommendations to standardize sample handling and patient preparation to reduce pre-analytical variability. Osteoporos Int. 2017;28(9):2541–2556. 10.1007/s00198-017-4082-4. 28631236

[10] Fisher A, Fisher L, Srikusalanukul W, Smith PN. Bone turnover status: classification model and clinical implications. Int J Med Sci. 2018;15(4):323–338. 10.7150/ijms.22747. 29511368 PMC5835703

[11] Clowes JA, Eghbali-Fatourechi GZ, McCready L, Oursler MJ, Khosla S, Riggs BL. Estrogen action on bone marrow osteoclast lineage cells of postmenopausal women in vivo. Osteoporos Int. 2009;20(5):761–769. 10.1007/s00198-008-0731-y. 18769961 PMC2842571

[12] Chung PL, Zhou S, Eslami B, Shen L, LeBoff MS, Glowacki J. Effect of age on regulation of human osteoclast differentiation. J Cell Biochem. 2014;115(8):1412–1419. 10.1002/jcb.24792. 24700654 PMC4096781

[13] Kim HN, Ponte F, Nookaew I, et al. Estrogens decrease osteoclast number by attenuating mitochondria oxidative phosphorylation and ATP production in early osteoclast precursors. Sci Rep. 2020;10(1):11933. 10.1038/s41598-020-68890-7. 32686739 PMC7371870

[14] Liu P, Gao Y, Luo P, et al. Glucocorticoid-induced expansion of classical monocytes contributes to bone loss. Exp Mol Med. 2022;54(6):765–776. 10.1038/s12276-022-00764-6. 35672449 PMC9256622

[15] Vahidi G, Moody M, Welhaven HD, et al. Germ-free C57BL/6 mice have increased bone mass and altered matrix properties but not decreased bone fracture resistance. J Bone Miner Res. 2023;38(8):1154–1174. 10.1002/jbmr.4835. 37221143 PMC10530360

[16] Rettedal EA, Ilesanmi-Oyelere BL, Roy NC, Coad J, Kruger MC. The gut microbiome is altered in postmenopausal women with osteoporosis and osteopenia. JBMR Plus. 2021;5(3):e10452. 10.1002/jbm4.10452. 33778322 PMC7990138

[17] Kameo Y, Miya Y, Hayashi M, Nakashima T, Adachi T. In silico experiments of bone remodeling explore metabolic diseases and their drug treatment. Sci Adv. 2020;6(10). 10.1126/sciadv.aax0938. PMC7060067 32181336

[18] Robling AG, Niziolek PJ, Baldridge LA, et al. Mechanical stimulation of bone in vivo reduces osteocyte expression of Sost/sclerostin. J Biol Chem. 2008;283(9):5866–5875. 10.1074/jbc.M705092200. 18089564

[19] Spatz JM, Wein MN, Gooi JH, et al. The Wnt inhibitor sclerostin is up-regulated by mechanical unloading in osteocytes in vitro. J Biol Chem. 2015;290(27):16744–16758. 10.1074/jbc.M114.628313. 25953900 PMC4505423

[20] Suda T, Takahashi N, Udagawa N, Jimi E, Gillespie MT, Martin TJ. Modulation of osteoclast differentiation and function by the new members of the tumor necrosis factor receptor and ligand families. Endocr Rev. 1999;20(3):345–357. 10.1210/edrv.20.3.0367. 10368775

[21] Nakashima T, Hayashi M, Takayanagi H. New insights into osteoclastogenic signaling mechanisms. Trends Endocrinol Metab. 2012;23(11):582–590. 10.1016/j.tem.2012.05.005. 22705116

[22] Charlebois DA, Balazsi G. Modeling cell population dynamics. In Silico Biol. 2019;13(1-2):21–39. 10.3233/ISB-180470. 30562900 PMC6598210

[23] Hill AV . The combinations of haemoglobin with oxygen and with carbon monoxide. I. Biochem J. 1913;7(5):471–480. 10.1042/bj0070471. 16742267 PMC1550542

[24] Kim YK, Kameo Y, Tanaka S, Adachi T. Capturing microscopic features of bone remodeling into a macroscopic model based on biological rationales of bone adaptation. Biomech Model Mechanobiol. 2017;16(5):1697–1708. 10.1007/s10237-017-0914-6. 28523374

[25] Fazzalari NL, Kuliwaba JS, Atkins GJ, Forwood MR, Findlay DM. The ratio of messenger RNA levels of receptor activator of nuclear factor kappaB ligand to osteoprotegerin correlates with bone remodeling indices in normal human cancellous bone but not in osteoarthritis. J Bone Miner Res. 2001;16(6):1015–1027. 10.1359/jbmr.2001.16.6.1015. 11393778

[26] Cao J, Venton L, Sakata T, Halloran BP. Expression of RANKL and OPG correlates with age-related bone loss in male C57BL/6 mice. J Bone Miner Res. 2003;18(2):270–277. 10.1359/jbmr.2003.18.2.270. 12568404

[27] Wijenayaka AR, Kogawa M, Lim HP, Bonewald LF, Findlay DM, Atkins GJ. Sclerostin stimulates osteocyte support of osteoclast activity by a RANKL-dependent pathway. PLoS One. 2011;6(10):e25900. 10.1371/journal.pone.0025900. 21991382 PMC3186800

[28] Tu X, Delgado-Calle J, Condon KW, et al. Osteocytes mediate the anabolic actions of canonical Wnt/β-catenin signaling in bone. Proc Natl Acad Sci U S A. 2015;112(5):E478–E486. 10.1073/pnas.1409857112. 25605937 PMC4321271

[29] Perkins SL, Gibbons R, Kling S, Kahn AJ. Age-related bone loss in mice is associated with an increased osteoclast progenitor pool. Bone. 1994;15(1):65–72. 10.1016/8756-3282(94)90893-1. 8024854

[30] Koshihara Y, Suematsu A, Feng D, Okawara R, Ishibashi H, Yamamoto S. Osteoclastogenic potential of bone marrow cells increases with age in elderly women with fracture. Mech Ageing Dev. 2002;123(10):1321–1331. 10.1016/S0047-6374(02)00071-4. 12297335

[31] Cao JJ, Wronski TJ, Iwaniec U, et al. Aging increases stromal/osteoblastic cell-induced osteoclastogenesis and alters the osteoclast precursor pool in the mouse. J Bone Miner Res. 2005;20(9):1659–1668. 10.1359/JBMR.050503. 16059637

[32] Kuroshima S, Al-Salihi Z, Yamashita J. Mouse anti-RANKL antibody delays oral wound healing and increases TRAP-positive mononuclear cells in bone marrow. Clin Oral Investig. 2016;20(4):727–736. 10.1007/s00784-015-1550-0. PMC4840226 26254598

[33] Anastasilakis AD, Makras P, Yavropoulou MP, Tabacco G, Naciu AM, Palermo A. Denosumab discontinuation and the rebound phenomenon: a narrative review. J Clin Med. 2021;10(1). 10.3390/jcm10010152. PMC7796169 33406802

[34] Boyce RW, Brown D, Felx M, et al. Decreased osteoprogenitor proliferation precedes attenuation of cancellous bone formation in ovariectomized rats treated with sclerostin antibody. Bone Rep. 2018;8:90–94. 10.1016/j.bonr.2018.03.001. 29955626 PMC6020110

[35] Sølling ASK, Harsløf T, Langdahl B. The clinical potential of romosozumab for the prevention of fractures in postmenopausal women with osteoporosis. Ther Adv Musculoskelet Dis. 2018;10(5-6):105–115. 10.1177/1759720X18775936. 29942362 PMC6009094

[36] Kim HN, Chang J, Shao L, et al. DNA damage and senescence in osteoprogenitors expressing Osx1 may cause their decrease with age. Aging Cell. 2017;16(4):693–703. 10.1111/acel.12597. 28401730 PMC5506444

[37] Kim HN, Chang J, Iyer S, et al. Elimination of senescent osteoclast progenitors has no effect on the age-associated loss of bone mass in mice. Aging Cell. 2019;18(3):e12923. 10.1111/acel.12923. 30773784 PMC6516158

[38] Piemontese M, Almeida M, Robling AG, et al. Old age causes de novo intracortical bone remodeling and porosity in mice. JCI Insight. 2017;2(17). 10.1172/jci.insight.93771. PMC5621920 28878136

[39] Farr JN, Xu M, Weivoda MM, et al. Targeting cellular senescence prevents age-related bone loss in mice. Nat Med. 2017;23(9):1072–1079. 10.1038/nm.4385. 28825716 PMC5657592

[40] Werner J . System properties, feedback control and effector coordination of human temperature regulation. Eur J Appl Physiol. 2010;109(1):13–25. 10.1007/s00421-009-1216-1. 19787369

[41] Saleem S, Teal PD, Howe CA, Tymko MM, Ainslie PN, Tzeng YC. Is the Cushing mechanism a dynamic blood pressure-stabilizing system? Insights from Granger causality analysis of spontaneous blood pressure and cerebral blood flow. Am J Physiol Regul Integr Comp Physiol. 2018;315(3):R484–R495. 10.1152/ajpregu.00032.2018. 29668325

[42] Hopkins BD, Pauli C, Du X, et al. Suppression of insulin feedback enhances the efficacy of PI3K inhibitors. Nature. 2018;560(7719):499–503. 10.1038/s41586-018-0343-4. 30051890 PMC6197057

[43] Becker NB, Gunther M, Li C, Jolly A, Hofer T. Stem cell homeostasis by integral feedback through the niche. J Theor Biol. 2019;481:100–109. 10.1016/j.jtbi.2018.12.029. 30579956

[44] Uhl P, Lowengrub J, Komarova N, Wodarz D. Spatial dynamics of feedback and feedforward regulation in cell lineages. PLoS Comput Biol. 2022;18(5):e1010039. 10.1371/journal.pcbi.1010039. 35522694 PMC9116666

[45] Frost HM . The mechanostat: a proposed pathogenic mechanism of osteoporoses and the bone mass effects of mechanical and nonmechanical agents. Bone Miner. 1987;2(2):73–85. 3333019

[46] Aarden EM, Burger EH, Nijweide PJ. Function of osteocytes in bone. J Cell Biochem. 1994;55(3):287–299. 10.1002/jcb.240550304. 7962159

[47] Burger EH, Klein-Nulend J. Mechanotransduction in bone—role of the lacunocanalicular network. FASEB J. 1999;13(9001):S101–S112. 10.1096/fasebj.13.9001.s101. 10352151

[48] Huiskes R, Ruimerman R, van Lenthe GH, Janssen JD. Effects of mechanical forces on maintenance and adaptation of form in trabecular bone. Nature. 2000;405(6787):704–706. 10.1038/35015116. 10864330

[49] Adachi T, Tsubota K, Tomita Y, Hollister SJ. Trabecular surface remodeling simulation for cancellous bone using microstructural voxel finite element models. J Biomech Eng. 2001;123(5):403–409. 10.1115/1.1392315. 11601724

[50] Pivonka P, Zimak J, Smith DW, et al. Model structure and control of bone remodeling: a theoretical study. Bone. 2008;43(2):249–263. 10.1016/j.bone.2008.03.025. 18514606

[51] Scheiner S, Pivonka P, Hellmich C. Coupling systems biology with multiscale mechanics, for computer simulations of bone remodeling. Comput Methods Appl Mech Eng. 2013;254:181–196. 10.1016/j.cma.2012.10.015.

