# Supplementary material for: Aging effects on osteoclast progenitor dynamics affect variability in bone turnover via feedback regulation
Source: JBMR Plus. 2024 Jan 4;8(1):ziad003. doi: 10.1093/jbmrpl/ziad003 (PMC11059999; doi:10.1093/jbmrpl/ziad003)
Supplement: ProgenitorJ_Supplemental_Materials_ziad003 [file progenitorj_supplemental_materials_ziad003.pdf]

Supplemental materials

Supplemental Figure S1

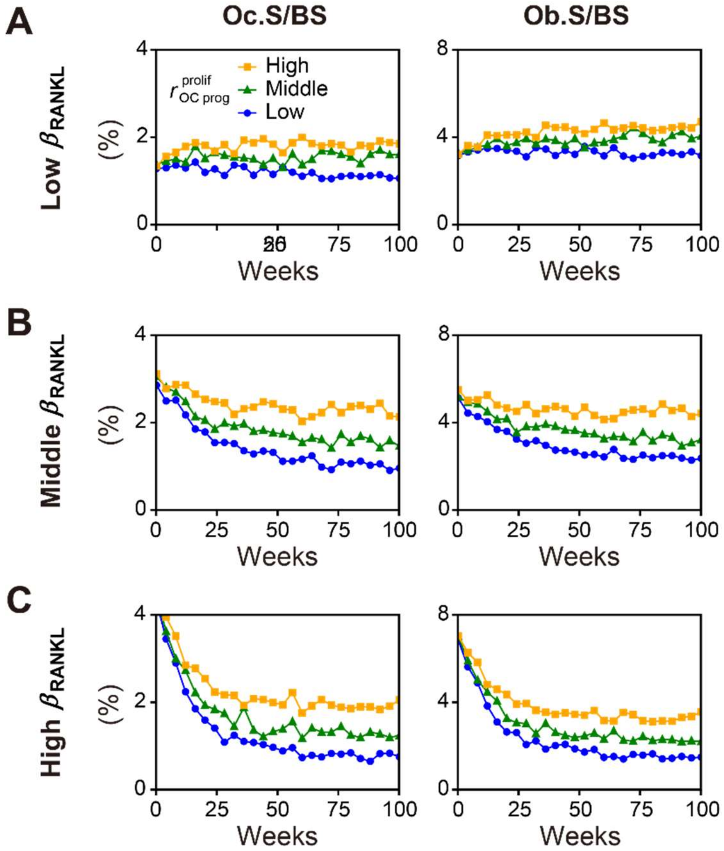

Fig. S1. Age-related enhancement of osteoclast progenitor proliferation maintains high bone turnover with osteoclast dominance. (A–C) Comparison of cell activities with aging effects on osteoclast progenitor proliferation among RANKL conditions. In each condition of β_{RANKL} (= 0.24, 0.28, and 0.32 pM/s), aging effects were set as a low, middle, and high rate of osteoclast progenitor proliferation $r_{\mathrm{OC\ prog}}^{\mathrm{prolif}}$ (= 0.002, 0.003, and 0.004 /mm^3/day). Oc.S/BS: osteoclast surface/bone surface. Ob.S/BS: osteoblast surface/bone surface.

Supplemental Tables

Table S1. Parameters associated with osteocyte mechanosensing and bone resorption/formation

Symbol	Description	Value	Unit
ρ_{ocy}	Number density of osteocytes	25000	mm^{-3}
σ_{max}	Maximum von Mises equivalent stress	20	MPa
σ_{min}	Minimum von Mises equivalent stress	1.0	MPa
l_L	Maximum distance for intercellular communication	50	μm
$\dot{M}_{\mathrm{r}}$	Bone resorption rate	–1.4	μm/day
$\dot{M}_{\mathrm{f}}$	Bone formation rate	0.56	μm/day

Table S2. Parameters associated with signaling molecules

Symbol	Description	Value	Unit
β_{SCL}	Maximum production rate of sclerostin	0.24	pM/s
β_{RANKL}	Maximum production rate of RANKL	0.24–0.32	pM/s
β_{Nrp1}	Maximum production rate of Nrp1	0.05	pM/s
P_{OPG}	Production rate of OPG	0.24	pM/s
P_{Sema3A}	Production rate of Sema3A	0.24	pM/s
P_{PlxnA}	Production rate of PlxnA	0.05	pM/s
k_{deg}	Degradation rate constant	0.005	1/s
D_{SCL}	Diffusion coefficient of sclerostin	5.58	μm²/s
D_{RANKL}	Diffusion coefficient of RANKL	5.32	μm²/s
D_{OPG}	Diffusion coefficient of OPG	4.49	μm²/s
D_{Sema3A}	Diffusion coefficient of Sema3A	3.62	μm²/s
k_{on}^{RO}	Association binding constant for RANKL-OPG binding	0.001	1/(pM・s)
k_{on}^{NP}	Association binding constant for Nrpl-PlxnA binding	0.01	1/(pM・s)
k_{off}^{NP}	Disassociation binding constant for Nrpl-PlxnA binding	0.1	1/s
k_{on}^{SNP}	Association binding constant for Sema3A and Nrpl-PlxnA complex binding	0.1	1/(pM・s)
k_{off}^{SNP}	Disassociation binding constant for Sema3A and Nrpl-PlxnA complex binding	0.1	1/s
K_{SCL}	Activation/inhibition constant for sclerostin — on RANKL	9.0	pM
	— on osteoblastogenesis	2.8	pM
	— on osteoblast apoptosis	2.8	pM
K_{RANKL}	Activation/inhibition constant for RANKL	18	pM
K_{SNP}	Activation/inhibition constant for Sema3A-Nrpl-PlxnA complex	200	pM
n	Hill coefficient	3.0	-

Table S3. Parameters associated with progenitor dynamics

Symbol	Description	Value	Unit
$r_{\mathrm{OC}}^{\mathrm{diff}}$	Differentiation rate of osteoclast	0.01	1/(mm³・day)
$r_{\mathrm{OB}}^{\mathrm{diff}}$	Differentiation rate of osteoblast	0.012	1/(mm³・day)
$r_{\mathrm{OC\ prog}}^{\mathrm{prolif}}$	Proliferation rate of osteoclast progenitor	0.002-0.004	1/(mm³・day)
$r_{\mathrm{OB\ prog}}^{\mathrm{prolif}}$	Proliferation rate of osteoblast progenitor	0.004	1/(mm³・day)
$N_{\mathrm{OC\ prog}}^{\mathrm{init}}$	Initial number density of osteoclast progenitor	100	1/mm³
$N_{\mathrm{OB\ prog}}^{\mathrm{init}}$	Initial number density of osteoclast progenitor	1000	1/mm³
$N_{\mathrm{OC\ prog}}^{\mathrm{max}}$	Maximum number density of osteoclast progenitor	300	1/mm³
$N_{\mathrm{OB\ prog}}^{\mathrm{max}}$	Maximum number density of osteoblast progenitor	1500	1/mm³
$K_{\mathrm{OC\ prog}}$	Activation constant for osteoclast progenitor	150	1/mm³
$K_{\mathrm{OB\ prog}}$	Activation constant for osteoblast progenitor	500	1/mm³
n	Hill coefficient	3.0	-
